# Towards Reproducible Descriptions of Neuronal Network Models

**DOI:** 10.1371/journal.pcbi.1000456

**Published:** 2009-08-07

**Authors:** Eilen Nordlie, Marc-Oliver Gewaltig, Hans Ekkehard Plesser

**Affiliations:** 1 Department of Mathematical Sciences and Technology, Norwegian University of Life Sciences, Aas, Norway; 2 Honda Research Institute Europe GmbH, Offenbach, Germany; 3 Center for Biomedical Computing, Simula Research Laboratory, Lysaker, Norway; 4 RIKEN Brain Science Institute, Wako-shi, Saitama, Japan; University College London, United Kingdom

## Abstract

Progress in science depends on the effective exchange of ideas among scientists. New ideas can be assessed and criticized in a meaningful manner only if they are formulated precisely. This applies to simulation studies as well as to experiments and theories. But after more than 50 years of neuronal network simulations, we still lack a clear and common understanding of the role of computational models in neuroscience as well as established practices for describing network models in publications. This hinders the critical evaluation of network models as well as their re-use.

We analyze here 14 research papers proposing neuronal network models of different complexity and find widely varying approaches to model descriptions, with regard to both the means of description and the ordering and placement of material. We further observe great variation in the graphical representation of networks and the notation used in equations. Based on our observations, we propose a *good model description practice*, composed of guidelines for the organization of publications, a checklist for model descriptions, templates for tables presenting model structure, and guidelines for diagrams of networks. The main purpose of this *good practice* is to trigger a debate about the communication of neuronal network models in a manner comprehensible to humans, as opposed to machine-readable model description languages.

We believe that the *good model description practice* proposed here, together with a number of other recent initiatives on data-, model-, and software-sharing, may lead to a deeper and more fruitful exchange of ideas among computational neuroscientists in years to come. We further hope that work on standardized ways of describing—and thinking about—complex neuronal networks will lead the scientific community to a clearer understanding of high-level concepts in network dynamics, and will thus lead to deeper insights into the function of the brain.

## Author Summary

Scientists make precise, testable statements about their observations and models of nature. Other scientists can then evaluate these statements and attempt to reproduce or extend them. Results that cannot be reproduced will be duly criticized to arrive at better interpretations of experimental results or better models. Over time, this discourse develops our joint scientific knowledge. A crucial condition for this process is that scientists can describe their own models in a manner that is precise and comprehensible to others. We analyze in this paper how well models of neuronal networks are described in the scientific literature and conclude that the wide variety of manners in which network models are described makes it difficult to communicate models successfully. We propose a *good model description practice* to improve the communication of neuronal network models.

## Introduction

Science advances human knowledge through learned discourse based on mutual criticism of ideas and observations. This discourse depends on the unambiguous specification of hypotheses and experimental procedures—otherwise any criticism could be diverted easily. Moreover, communication among scientists will be effective only if a publication evokes in a reader the same ideas as the author had in mind upon writing [1].

Scientific disciplines have over time developed a range of abstract notations, specific terminologies and common practices for describing methods and results. These have lifted scientific discourse from handwaving arguments about sloppily ascertained observations to precise and falsifiable reasoning about facts established at a well-defined level of certainty. Well chosen notation and systematization, from Linné's classification of flora and fauna, via the periodic system of the elements to Feynman diagrams have widened the minds of scientists and continue to induce new discoveries.

Matrix notation provides an illustrative example of the power of notation. Consider a system of three differential equations
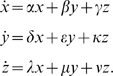
(1)Defining 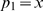, 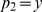, 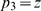 and 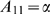, 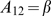, etc., we can write this more compactly as
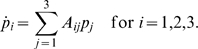
(2)Introducing matrix notation simplifies this further to
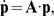
(3)with multiple advantages: the equation is much more compact, since the summing operation is hidden, as well as the system size; most importantly, the equation is essentially reduced to a simple multiplication. This invites further exploration.

From the study of one-dimensional differential equations, we know that
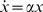
(4)has the solution
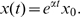
(5)Comparing the shape of Eq. 4 to Eq. 3 immediately suggests the following solution to Eq. 3
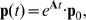
(6)with the formal definition
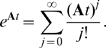
(7)This formal solution can be made rigorous, and underlies the exact integration method [2]. It is hard to see how the inspiration to write down a solution such as Eq. 3 might have arisen from the original form of the differential equations in Eq. 1.

Note that even though the notion and notation of vectors and matrices is more abstract and, thus, more compact than the original formulation of Eq. 1, it does not lose any detail. The variables 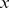, 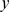, and 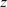 from the original system Eq. 1 are still present, not as separate entities, but as components of the vector 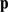. The specific combinations of additions and multiplications are embedded in the multiplication rule for vectors. To arrive at the concise notation of Eq. 2 we must introduce the new mathematical concept of vector spaces. This example illustrates how scientific notation progresses together with scientific concepts.

Computational neuroscience lags behind mathematics and other fields of science in standardization, expressiveness and power of notation. We assess here the current scientific practice of describing computational models of the brain. We focus on network models built from large numbers of rather simple neurons with an aim to test hypotheses on aspects of brain function. Specifically, we study 14 papers chosen mainly from visual neuroscience [3]–[16]; see Table 1 for a brief summary of the models. Our selection of papers is by no means comprehensive, although we have attempted to cover past as well as current work, and to include a range of different approaches to the description of neuronal network models.

**Table 1:** Papers analyzed in this study.

Reference	Abbr.	Description
Brunel [3]	B	Unordered network of two populations of integrate-and-fire neurons with current-injecting synapses; random external input.
Destexhe et al. [4]	D	One-dimensional network with two layers of point neurons with several ionic currents and conductance based synapses.
Haeusler and Maass [5]	HM	Unordered six-population model of Hodgkin-Huxley-type neurons with conductance-based synapses with short-term dynamics.
Hayot and Tranchina [6]	HT	Two-dimensional network with three populations of firing-rate neurons; spatiotemporally patterned input.
Hillenbrand and van Hemmen [7]	HvH	Model of corticogeniculate loops that tests if the visual cortex controls the spatiotemporal structure of cortical receptive fields via feedback to the lateral geniculate nucleus.
Izhikevich and Edelman [8]	IE	“Whole brain” model covering several brain areas, each composed of layered two-dimensional networks of oscillator neurons with plastic, conductance-based synapses.
Kirkland and Gerstein [9]	KG	Two-dimensional model of three layers of integrate-and-fire neurons with conductance-based synapses driven by spatiotemporally pattered stimuli.
Lumer et al. [10]	L	Two-dimensional model of ten layers, with two neuron populations per layer; integrate-and-fire neurons with conductance-based synapses.
Mariño et al. [11]	M	Two-dimensional model of two layers of Hodgkin-Huxley-type neurons with conductance-based synapses.
Saam and Eckhorn [12]	SE	Two-dimensional model of two layers of pulse-coding neurons.
Tao et al. [13]	TA	Two-dimensional two-layer model of integrate-and-fire neurons with conductance based synapses.
Troyer et al. [14]	TR	Two-dimensional network model with two populations of conductance-based integrate-and-fire neurons.
Vogels and Abbott [15]	VA	Unordered and one-dimensional networks of integrate-and-fire neurons.
Wielaard and Sajda [16]	WS	Two-dimensional two-layer model of integrate-and-fire neurons with conductance based synapses.

The table gives a brief overview of the type of model studied and assigns an abbreviation to each paper for reference in other tables.

A central motivation for our work is that sharing of materials, methods, and data in the life sciences has received increased attention in recent years, to a large part driven by developments in molecular biology. The UPSIDE (*uniform principle for sharing integral data and materials expeditiously*) doctrine proposed by the *Committee on Responsibilities of Authorship in the Biological Sciences* of the National Academies of Science (USA) defines the most comprehensive set of rules for data sharing [17] and has been adopted by several leading journals [18],[19]. Sharing of experimental data has received increasing attention in the neurosciences recently [20]–[23].

Sejnowski et al. [24] gave a fine account of the role of modeling in neuroscience 20 years ago, when computational neuroscience as a field just “took off”. They characterized models as “provisional framework[s] for organizing possible ways of thinking about the nervous system.” Since then, modeling activity has multiplied, but reflection about the modeling process has hardly kept up.

Computational neuroscientists are only now beginning to pay increasing attention to the role of models and simulations, as well as preconditions for the successful exchange of models, as witnessed by recent workshops [25],[26], collaborative reviews of simulation software [27], and the development of software providing common interfaces [28],[29] and run-time interaction of simulations on different simulators [30]. Most of these discussions have been rather technical, though, and little attention has been paid to the intellectual gain as part of the modeling process or to the issue of how to convey models and simulations best in scientific publications. Researchers in ecology, systems biology and physiome modeling appear to be significantly ahead in these issues [31]–[37]. Indeed, De Schutter [38] recently suggested that computational neuroscience has much to learn from systems biology.

### The nature of neuronal network models

Philosophers of science have yet to develop a robust definition and interpretation of models and simulations [39]–[42]. Most of that debate focuses on models in physics, but Peck [31] gives an interesting review of models and simulations in ecology, while Aumann [32] thoroughly discusses requirements of successful modeling of ecological systems; Wooley and Lin [43] give an overview of modeling and simulation in biology. The only comparable assessment of the role of models and simulations in computational neuroscience is part of a book chapter by Clark and Eliasmith [44]. A recent appraisal of the role of models in neuroscience [45]–[47], based on a general reappraisal of the role of computational models by Humphreys [48], has mostly focused on connectionist models.

We shall not attempt to provide a general analysis of models and simulations in computational neuroscience here. Our aim is more practical: to promote standards for the description of neuronal network models in the literature, to further sharing of knowledge and facilitate critique. Thus, our focus is narrower yet than that of Eliasmith and Anderson [49], Ch. 1.5, who proposed a “Methodology” of neural engineering. For our purposes, we adopt a quite restricted working definition of a model:

A neuronal network model is an explicit and specific hypothesis about the structure and microscopic dynamics of (a part of) the nervous system.

Several aspects of this definition deserve note:

- The model must be *explicit*, i.e., all aspects of the model must be specified.
- The model must be *specific*, i.e., all aspects must be defined so detailed that they can be implemented unequivocally.
- The model specifies the *structure* (placement and type of network elements; source, target and type of connections) and *dynamics of components* (ion channels, membrane potential, spike generation and propagation).
- The model does *not* describe the dynamics of the model as a whole, which is an emerging property of the model.

The model is first of all a mental model formed in the brain of a researcher. It is her hypothesis about the function of a part of the brain. Heinrich Hertz expressed this idea first in his textbook “Prinzipien der Mechanik” in 1894:

“We make for ourselves internal images or symbols of the external objects, and we make them in such a way that the consequences of the images that are necessary in thought are always images of the consequences of the depicted objects that are necessary in nature Once we have succeeded in deriving from accumulated previous experience images with the required property, we can quickly develop from them, as if from models, the consequences that in the external world will occur only over an extended period or as a result of our own intervention.” (cited from [40]).

Scientific progress depends critically on the ability of neuroscientists to communicate models, i.e., hypotheses, among each other: When Anna presents her model to Bob and Charlie—will both build the same mental model in their minds as Anna? Or will some nuances be lost, some aspects interpreted differently, some parts misunderstood? Only a precise, unambiguous notation for models will allow Anna, Bob and Charlie to discuss their individual understandings of the model and thus to truly share models. Efficient communication dictates that scientists should use a common notation to describe their models, as it is demanding to thoroughly acquaint ourselves with any advanced notation.

It is tempting to consider implementations of neuronal network models in a specific simulator software as a sufficient model description, as it is explicit, specific and describes structure and dynamics. We believe this to be a fallacy. Implementations come most often in the form of scripts or computer programs, which tend to be difficult to reverse engineer: It is simply not possible to infer the overall network structure from the bits and pieces of a large script. Secondly, most simulation scripts rely on properties hidden in a simulator, which may even change as a simulator evolves over time. Translating a given implementation first to a mental model and then to a second simulator software for independent testing, opens for errors in both translation steps. We believe that while scientific productivity benefits from sharing simulation code through repositories such as ModelDB [50] and standard languages such as NeuroML [51], implementations do not fill the need for precise human-readable model descriptions in the scientific literature. Based on experiences in systems biology, Wimalaratne et al. [36] stress that it is crucial to identify biophysical concepts as logical abstractions in order to create meaningful and re-usable model implementations.

It is also worth mentioning that the translation of a mathematical model into a computer program is lossy and irreversible. The translation is lossy due to the finite precisions of computers. For example, most real numbers cannot be represented on a computer. This is obviously problematic in the analysis of chaotic systems where small errors have a big influence on the state trajectories of the system. The translation is generally not reversible, because the commonly used programming languages are not accessible to formal analysis. It is generally not even possible to prove that a function, implemented in a common language such as C++, is correct. In some cases, one may even have to add equations to models in the computer implementation to preserve stability and obtain results in agreement with experimental observation [42],[52].

While mathematical model descriptions can be treated with formal methods, their computer implementations generally cannot. This means that if we want to validate the claims about a model, we must start from the description in the scientific publication. If we start from the model implementation of the authors, we can *never* refute that the model may be faulty or doing something entirely different than what was claimed in the publication. Taking a given implementation of a model or hypothesis and simply executing it again does *not* constitute independent testing, nor does it fulfill the criterion of *falsifiability*: the same program run twice should yield identical results.

## Methods

We shall now sketch key aspects of neuronal network model descriptions: what is described where and by what means in the computational neuroscience literature? This will introduce the conceptual framework for the subsequent analysis of the papers given in Table 1.

### Components of model descriptions

A complete model description must cover at least the following three components: (i) The *network architecture*, i.e., the composition of the network from areas, layers, or neuronal sub-populations. (ii) The *network connectivity*, describing how neurons are connected among each other in the network. In most cases, connectivity will be given as a set of rules for generating the connections. (iii) The *neuron and synapse models* used in the network model, usually given by differential equations for the membrane potential and synaptic currents or conductances, rules for spike generation and post-spike reset. Model descriptions should also contain information about (iv) the *input (stimuli)* applied to the model and (v) the *data recorded* from the model, just as papers in experimental neuroscience do, since a reproduction of the simulations would otherwise become impossible.

### Means of model descriptions

Neuronal network models are usually described by a combination of five means: prose (text), equations, figures, tables and pseudocode. We shall discuss these in turn.

*Prose* is a powerful means of communicating ideas, intentions and reasons. It is flexible and, if used carefully, precise. Unfortunately, prose can easily—often unintentionally—become ambiguous. Previous knowledge and ideas in the mind of the reader will shape the reader's understanding of a textual description of a model and may lead to misunderstandings. Prose that strives to be strictly unambiguous and provide all required detail, on the other hand, will often be difficult to read.

*Mathematical notation (equations)* is compact and unambiguous. Suitably chosen notation compresses complex relationships in concise expressions, which allow for further manipulation in our mind, as illustrated by the matrix exponentiation in the Introduction. The now common mathematical notation emerged alongside the great scientific achievements of Newton, Leibniz and others between the 17^th^ and 19^th^ century [53],[54]. Unfortunately, not all mathematical notation is understood easily, and variations in notation, as is common in computational neuroscience (cf. Table 2), can present serious obstacles to effective communication.

**Table 2:** Membrane potential equations for some papers using conductance-based neurons.

Destexhe et al. [4, Eq. 2]	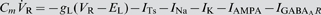
Lumer et al. [10]	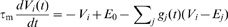
Tao et al. [13, Eq. 1]	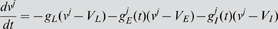
Vogels and Abbott [15, Eq. 2]	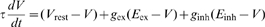

The model by Destexhe et al. is a Hodgkin-Huxley style neuron, all others are integrate-and-fire neurons.

*Figures* communicate the architecture and connectivity of network models well, since vision is the dominating sense in most humans. Most readers will first scan the figures in a paper to get an overview of what the paper is about, using figure captions as a guide, and read the full text of the paper only later. Thus, figures and captions will shape the initial idea a reader forms about a neuronal network model, and the ideas thus established may be difficult to correct through textual description. Specifying complex networks precisely in figures can be difficult, and disciplines depending strongly on exact diagrams, such as mechanical and electrical engineering, have developed precise standards for such diagrams (see, e.g., [55]). Systems biologists have yet to arrive at a definite standard for depicting their models, but they at least have an open debate about graphical representations [56]–[59].

*Tables* are a useful means of organizing data, especially model parameters. Data presented in table form is far more accessible than data dispersed throughout a text, facilitating, e.g., comparisons of parameter choices between different papers and proof-reading of simulation scripts against papers.

*Pseudocode* is often used to present algorithms in concise, human readable form, without resorting to a specific programming language. It will be an efficient means of communication only if the pseudocode notation is sufficiently well established to be unambiguous.

### Placement of model descriptions

The placement of model descriptions within a scientific publication depends on the focus of the paper and the journal it is published in. Traditionally, model descriptions were either given in the *body text* of a paper, or in an *appendix*. It has now become common to give only brief model overviews in the paper itself, and to relegate detailed model descriptions to *supplementary material* published online, or even to place simulation code online in community *repositories* such as ModelDB.

## Results

We will now analyze model descriptions in the 14 papers listed in Table 1. We study the placement of model descriptions in publications first, followed by a general discussion of the means of description used. We will then investigate in more detail how specific aspects of models are described. Finally, we propose a *good model description practice*.

### Placement of description

**Figure 1:**
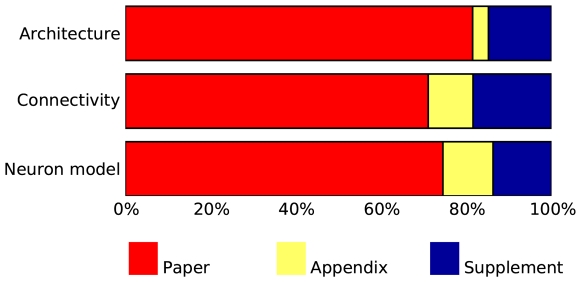
Placement of description in papers surveyed. Bar graphs show the percentage of papers describing (from top to bottom) model architecture, model connectivity and neuronal dynamics in the body text of the paper, the appendix, and in supplementary material. Many papers spread descriptions over several locations and are thus counted in several categories. For detailed data, see supporting material Tables S1, S2 and S3.

Figure 1 summarizes the placement of the description of architecture, connectivity and neuron and synapse models, respectively, across all papers; for details, see Tables S1, S2 and S3 in the Supporting files. All papers present at least an overview of the model they investigate in the main body of the paper. Details are frequently provided in supplementary material available online, especially in more recent papers; appendices are used to a lesser degree. Model descriptions in some papers are incomplete in the sense that the authors refer to other publications for details of neuronal dynamics in particular.

Within the body text of the paper, model descriptions were placed in the “Methods” sections in 10 of the 14 papers surveyed, even though the neuronal network model is in itself a product of significant scientific analysis and synthesis [32]. As such, it would rather belong in the “Results” section of a paper. Whether the placement of the model description in the “Methods” section genuinely reflects the way in which authors perceive their models, or rather is a consequence of editorial policies shaped by “wet” neuroscience, is not clear at present. It is interesting to note in this context that papers in theoretical physics generally do not follow the strict “methods-results-discussion” pattern.

We would like to point out two interesting aspects of the placement of model descriptions. First, the text of a paper manuscript, including the appendix, undergoes thorough peer review and copy editing, ensuring high standards in content and presentation. It is not, at present, clear whether all material published as supplementary material receives the same scrutiny in the review process; it is often not copy-edited to the same standards as the paper proper. Second, source code published in community repositories represents an implementation of a model, not the model itself [52]. It can thus serve only as a service to the community to facilitate code-reuse, but not to communicate the content of the model proper.

Incidentally, none of the 14 papers surveyed here describes re-use of neuronal models available in repositories, such as ModelDB [50]. Nor does any paper mention that the source code for the model implemented in the paper was made available to the community, even though models from several papers are at present available from ModelDB [4],[5],[15]. In recent years, though, there appears to be a slowly growing trend to explicitly reference and re-use existing models from ModelDB; see http://senselab.med.yale.edu/modeldb/prm.asp for an up-to-date list (Michael Hines, personal communication).

### Means of model descriptions

**Figure 2:**
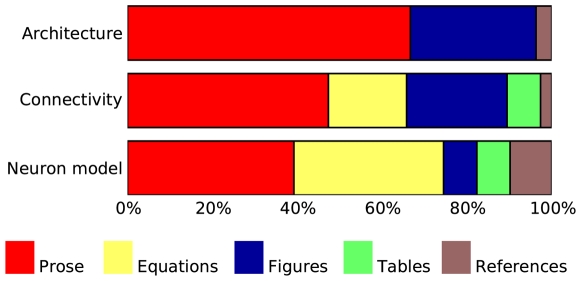
Use of different means of description in papers surveyed. Bar graphs show the percentage of papers describing (from top to bottom) model architecture, model connectivity and neuronal dynamics using prose, equations, figures, tables, and references. Many papers combine several means for one purpose and are thus counted in several categories. For detailed data, see supporting material Tables S1, S2, S3.

Figure 2 shows that equations are mostly used to describe the dynamics of model neurons, while connections are most often presented in a combination of prose and figures, occasionally in form of pseudocode. We will review the quality of these descriptions in detail below. Table 3 shows how parameters are presented in papers. It regrettably indicates that too few authors make parameters easily accessible in tables.

**Table 3:** Presentation of parameters.

All	Most	Some	None
—	IE, KG, L, SE	HM	B, D, HT, HvH, M, TA, TR, VA, WS

The table shows the papers presenting all, most, some or none of their parameters in tables. See Table 1 for paper abbreviations.

Network model descriptions in the literature show no consistent order of description. Among the papers surveyed here, six begin with a description of the neuron models and then proceed to network architecture, seven papers use the opposite order, while one paper mixes the description of neurons and network. We find the latter option least useful to the reader.

Authors differ greatly in their efforts to anchor their models in empirical data. Destexhe et al. [4] go to great lengths to justify the design of their neuron and synapse models with respect to the neurophysiological literature. They thus provide the *synthesis document* proposed by Aumann [32] as the basis of any modeling effort. Unfortunately for those readers who want to investigate the resulting model, though, model description and justification are tightly intertwined in the terse methods section, making it quite demanding to extract the model description as such.

Among all papers surveyed here, only Destexhe et al. [4] and Izhikevich and Edelman [8] show responses of individual synaptic conductances and individual neurons to test stimuli, while all other authors only show responses of the entire network. This means that researchers who attempt to re-implement a model and find themselves unable to reproduce the results from a paper, will not be able to find out whether problems arise from neuron model implementations or from a wrong network setup.

We will now analyze in detail which difficulties arise in describing a network model, considering in turn network architecture, connectivity, and neuron models, and point out examples of good descriptions.

### Network architecture

Descriptions of network architecture become challenging as network complexity increases. Networks with a small number of populations, random connectivity and no spatial structure are easily described in a few lines of prose, as in Brunel's paper [3]. A combination of prose and simple figures is usually sufficient to describe architecture of networks composed from a small number of one- or two-dimensional layers of individual neurons; examples are Destexhe et al. [4] and Kirkland and Gerstein [9].

Complex models spanning several brain areas with detailed spatial, layered, and functional substructure, such as Lumer et al. [10] and Izhikevich and Edelman [8], are more challenging to describe. Authors generally adopt a top-down approach, giving first an overview of the brain areas involved, before detailing the structure of the individual areas. In models of systems with clearly defined signal flow, areas are often visited in the predominant order of signal flow [6],[7],[14], while others present the more complex cortical structures before descending to subcortical structures [8],[10].

**Figure 3:**
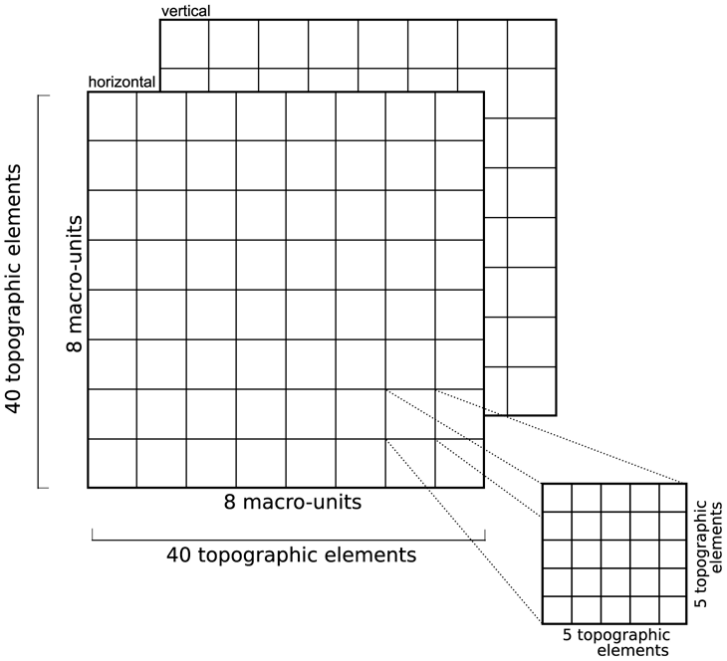
Interpretation of Lumer [10] model architecture. The most parsimonious interpretation of the description of the primary visual cortical area Vp given by Lumer *et al*, is as two layers of 40×40 topographic elements, representing horizontal and vertical orientations, respectively.

The most detailed explicit model studied here is the thalamocortical model presented by Lumer et al. [10]. The description of the cortical areas in this model (Vp and Vs), while complete, lacks in our opinion the clarity desirable of a good model description, and may thus help to identify rules for ideal model descriptions. For one, discussions on model design and properties are embedded in the model description, e.g., the reduction of a total of 32 “combinations of response selectivities” to just two included in the model, and a comparison of the number of neurons in the model to that found in animals. We believe that design decisions and model review should be kept separate from the model description proper for the sake of clarity, since they are independent intellectual endeavours [32]. Second, Lumer et al. mix different views of their layer architecture without providing sufficient guidance to the reader. They begin by describing the Vp layer as a grid of 8×8 macro-units, with two “selectivities within a macro-unit”, each containing “a collection of 5×5 topographic elements, each of which corresponded to a contiguous location in retinal space”, before proceeding to state that “[t]opographic elements in Vp were organized in maps of 40×40 elements for each of the two modeled orientation selectivities.” We find it difficult to interpret this description unambiguously. We are in particular in doubt about the localization of macro-units and topographic elements in retinal space. In our view, the most parsimonious interpretation is as follows: 5×5 topographic elements placed in each of 8×8 macro-units result in a grid of 40×40 topographic elements.” This interpretation is sketched in Fig. 3.

Another interesting aspect is that model composition is often described from a perspective orthogonal to the description of connections. Lumer et al. [10], e.g., present the primary thalamus and cortex as grids of 40×40 topographical units, each containing an excitatory and an inhibitory neuron (thalamus) and a microcolumn composed of 10 neurons organized in three laminae (cortex). Connections are then described by looking at this architecture from an orthogonal perspective: Thalamus is described as two layers, one of excitatory and one of inhibitory neurons, while cortex is split into six layers, one of excitatory and one of inhibitory neurons for each of the three laminae in the model. We believe that it may be more sensible to base the model description on the perspective used in defining connections, as connectivity is the central aspect of a network model.

Izhikevich and Edelman [8] present a significantly more complex model, covering the entire human cortex and thalamus. Concerning the spatial placement, they only state that “[n]euronal bodies are allocated randomly on the cortical surface, whose coordinates were obtained from anatomical MRI.” No further information is given on how MRI measurements were converted to neuron densities in space. Thus, even if one had access to MRI data of the human brain, it would be difficult to reproduce the neuron distribution investigated by Izhikevich and Edelman. In such cases it would be advantageous to either use datasets available from community databases or to make data available to others.

Figures of network architecture vary widely between papers. We will discuss them in the following section together with connections.

### Connections

Describing the connections well is the most challenging task in presenting a neuronal network model. For networks with random connections and no spatial structure, connectivity is easily described in a few sentences [3]. Haeusler and Maass [5] additionally represent connection strengths and probabilities in a figure; this works well for their six-population model. If yet more populations were involved, such a figure would soon become cluttered, and it becomes more useful to present connection parameters in tables , cf. supplementary material in ref. [8]. Even in these simple networks, care must be taken to specify details:

- May neurons connect to themselves?
- May there be multiple connections between any pair of neurons?
- Are connection targets chosen at random for a fixed sender neuron (divergent connection), senders chosen at random for fixed target (convergent connection), or are sender and receiver chosen at random for each connection?

Few authors are explicit on all these points, although these choices may have significant consequences for network dynamics (Tom Tetzlaff, personal communication; see also Kriener et al. [60]).

Models incorporating spatial structure have more complex connection patterns, which we will call *topographic* connections, since they usually describe the spatial distribution of connection targets relative to the spatial location of the sending neuron, i.e., connections are typically described as divergent connections. In most cases, connections have a random component: they are created with a certain probability. In simple cases, such as Kirkland and Gerstein [9], connections are made to neurons in a rectangular mask with equal probability. In more complex models, connection probability depends on the relative locations of the neurons that are candidates for a connection, e.g., [10],[11]. Unfortunately, few authors provide the equations for these probability functions; Mariño et al. [11] is a laudable exception. It is somewhat paradoxical if papers present long tables of parameters for these connection probability functions, but do not provide the equation into which these parameters enter.

Mariño et al. [11] are the only authors who explicitly discuss self-connections (in their supplementary material), and as far as we can see, no authors have discussed whether multiple connections between any two neurons may be created. Another neglected issue is precisely how probabilistic connections are created. The following approach seems to be implied: For each pair of neurons from the sender and target population, a connection is created if a random number is smaller than the connection probability for the pair. But one might equally well determine the total number of connections to be made first, and then distribute the connections according to the spatial probability profile [61]. Such schemes offer significant performance gains [62]. A complete specification of the connection algorithm should thus be given.

Among the papers surveyed, Izhikevich and Edelman [8] has by far the most complex connectivity and the authors go to great lengths to present gray-matter connectivity in figures, tables, and prose. Alas, some information appears to be missing: It is not clear from the text exactly how connections are distributed within the axonal spans, and how they are distributed across dendritic compartments of neurons with more than one compartment in a cortical layer. We have also been unable to find specific information on how synaptic weights and delays were assigned to connections. Finally, no details are provided about the white-matter (long-range) connections, which were based on diffusion-tensor imaging (DTI) data. Without access to the DTI data it is thus impossible to re-implement the model presented.

**Figure 4:**
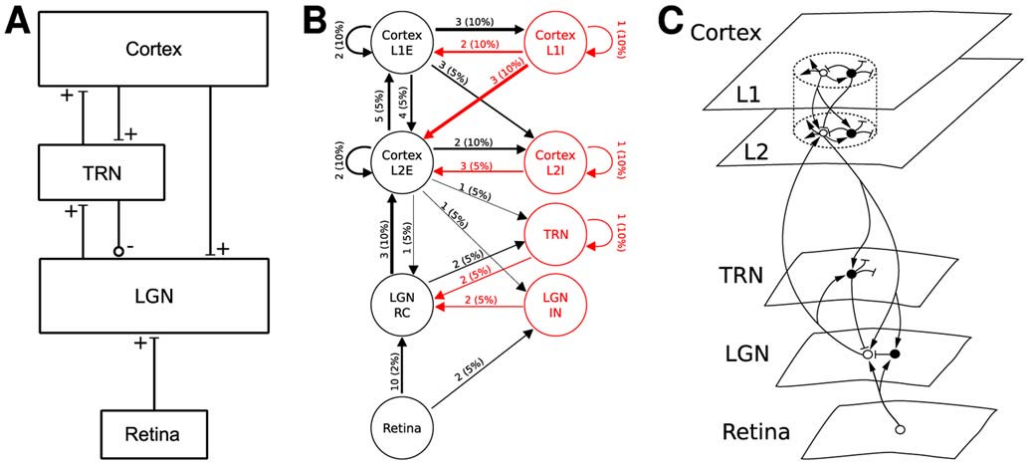
Diagram styles for network models. Diagrams of a model of the thalamocortical pathway drawn using diagram styles from (A) Hayot and Tranchina [6], Fig. 2, (B) Haeusler and Maass [5], Fig. 1, and (C) Lumer et al. [10], Fig. 1. Numbers on arrows in B mark connection weight and probability of connection, while line width represents the product of the two. In C, open circles show excitatory, filled circles inhibitory neurons. The model depicted is loosely based on Einevoll and Plesser [63, Fig. 3], but the differentiation into two cortical layers, each with excitatory and inhibitory subpopulations, in B and C, as well as the connection weights and probabilities, have been added here for the purpose of illustration.

Paper authors draw network diagrams in quite different ways, both in the overall style of their diagrams and in use of symbols. Figure 4 shows network diagrams of a model loosely based on Einevoll and Plesser [63], Fig. 3, drawn in the style of three of the papers surveyed here. The diagram in the style of Hayot and Tranchina [6] (Fig. 4A) gives a reduced but clear overview of the overall architecture of the model; it provides no details. The style of Haeusler and Maass [5] (Fig. 4B) carries most information, with weights and probabilities shown next to connection lines, and line widths proportional to the product of weight and probability. Figure 4C, which imitates the style of Lumer et al. [10], is rather illustrative: it provides no quantitative information and the structure of the connectivity is less prominent than in the other two figures; on the other hand, it is the only figure hinting at the spatial structure of the network. Interestingly, all three diagram styles use different ways of marking excitatory and inhibitory connections: bars vs circles, black vs red, and arrows vs bars. Indeed, bars at the end of connection lines mark excitatory connections in Hayot and Tranchina's style, but inhibitory connections in the style of Lumer et al, nicely illustrating the lack of standards in the field.

Izhikevich and Edelman [8] have illustrated their brain model using diagrams presenting significantly more detail than in the diagrams shown in our Fig. 4. Unfortunately, we cannot reproduce Figures 2 and 8 from the supplementary material of the paper by Izhikevich and Edelman here due to copyright issues; the figures are available on the internet at http://www.pnas.org/content/105/9/3593.figures-only and http://www.pnas.org/content/105/9/3593/suppl/DC1, respectively. Their diagrams, though, provide so much detail of interest to the re-implementer, that the reader will have difficulty to form a clear conceptual model from the diagram. This is in many ways the curse of complex models as the following analogy may illustrate: when a physicist or electrical engineer sees a diagram of an RLC circuit, she will intuitively “see” the circuit oscillate. When presented with the complete wiring diagram for a modern analog radio receiver, though, it is hardly likely she will “hear the music”. The figure in the style of Haeusler and Maass [5] takes a middle ground. Since the individual populations are homogeneous, they can be represented by one circle each, with annotated lines providing information about connection structure *and* parameters. By marking connection strength through line width and differentiating excitation and inhibition by line color, the figure appeals quite directly to our intuition. It is clear, though, that any further populations would increase the complexity of the diagram to the point of illegibility.

There is no established standard for the order in which connections within a network are described. Some authors proceed from local connectivity (e.g., intracortical intralaminar) towards global connectivity [10]. Others rather follow the signal flow through the network, from retina via LGN to cortex, e.g., Kirkland and Gerstein [9], Hayot and Tranchina [6], and Troyer et al. [14].

### Neuron and synapse models

Neuron and synapse models are commonly described by a mixture of prose and equations, cf. Fig. 2; tables are used inconsistently to present parameters, see Table 3. Some authors do not provide complete model specifications in their paper, but rely heavily [4] or even entirely [5] on references to earlier work. While the desire to avoid repetition is understandable, we believe that authors here walk a thin line toward incomprehensibility, especially if the models used are spread over three or more publications. Even though the re-use of neuron model implementations provided in repositories such as ModelDB may save effort and contribute to a standardization in the field, none of the papers we studied made use of available model implementations—or the authors failed to point out that they did.

Table 2 shows the membrane potential equations found in several papers and demonstrates that there is a reasonable amount of variation in the way this central equation is written down. There is in particular no widespread agreement on whether to include the membrane capacitance 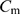 explicitly in the equation or rather to subsume it in a membrane time constant 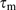. Some authors, such as Tao et al. [13], even chose to normalize the membrane potential equation by defining 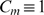. Yet greater variation is found in the representation of synaptic currents. This means that phrases such as “we use the standard equations for integrate-and-fire neurons”, which are not uncommon in the literature, are essentially meaningless, since there are no established “standard equations” for integrate-and-fire neurons.

Spike generation and detection, including subsequent reset and refractory behavior, are usually described in prose, sometimes with interspersed equations. “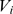 was reset to … 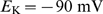, when it exceeded a threshold of … −51 mV …, at which point a spike was recorded, and relayed …,” is a typical formulation [10]. Unfortunately, it does not state precisely how threshold crossings are detected, which times are assigned to spikes, or when exactly the reset is executed. All these issues can have significant consequences for network dynamics [64]–[66].

### Good model description practice: a proposal

The previous sections have documented a wide variety of approaches to model descriptions in the literature. We believe that this variety is detrimental to the field, as it makes it difficult to communicate neuronal network models correctly and efficiently. At the same time, we believe that the field of computational neuroscience is too young to establish exacting standards for model descriptions. We will return to this problem and its various causes in the discussion. As a middle road, we propose to establish a *good model description practice* for the scientific literature. We will refer to it as “good practice” below for brevity. Some of our suggestions are motivated by a recent analysis of modeling techniques in ecology [32], but see also [49].

We propose a practice with the following elements:

1. *Guidelines for the organization* of a model description in a publication.
2. *Checklists for model descriptions* helping authors to present all required information in a useful order.
3. *Templates for tables* describing the essential aspects and components of a model in a compact, easily accessible manner.
4. *Guidelines for diagrams* visualizing neuronal network models.

**Figure 5:**
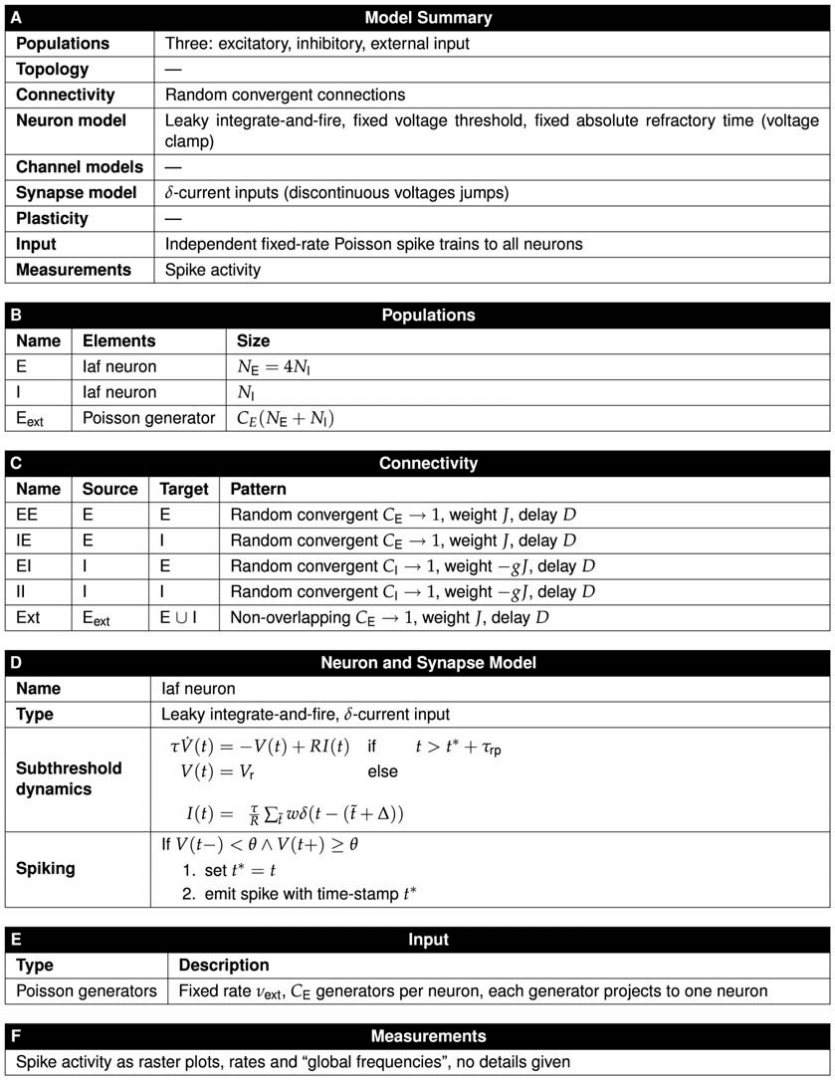
Tabular description of Brunel model [3]. The model is summarized in panel A and detailed in panels B–F.

**Figure 6:**
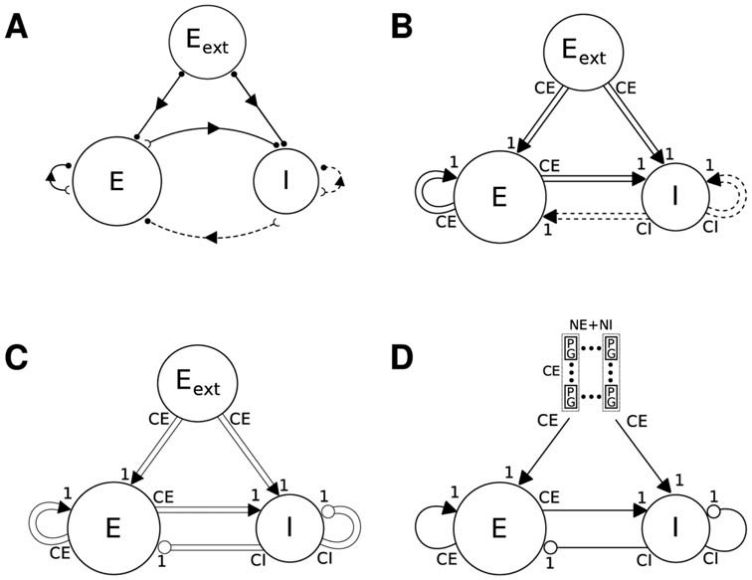
Alternatives for diagrams of simple network models (Brunel [10]). (A) Excitatory connections shown by full lines, inhibitory by dashed lines. Lines beginning with open semicircle and ending in filled circle indicate random convergent connections. (B) Double lines represent multiple connections, solid/dashed marks excitatory/inhibitory connections. Multiplicity of connections marked at line ends. (C) Same as B, but inhibitory connections marked with circles on target side instead of dashed lines. (D) Same as C, but displaying explicitly that there are 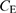 external Poisson inputs (PG) to each neuron, and single lines are used instead of double lines.

**Figure 7:**
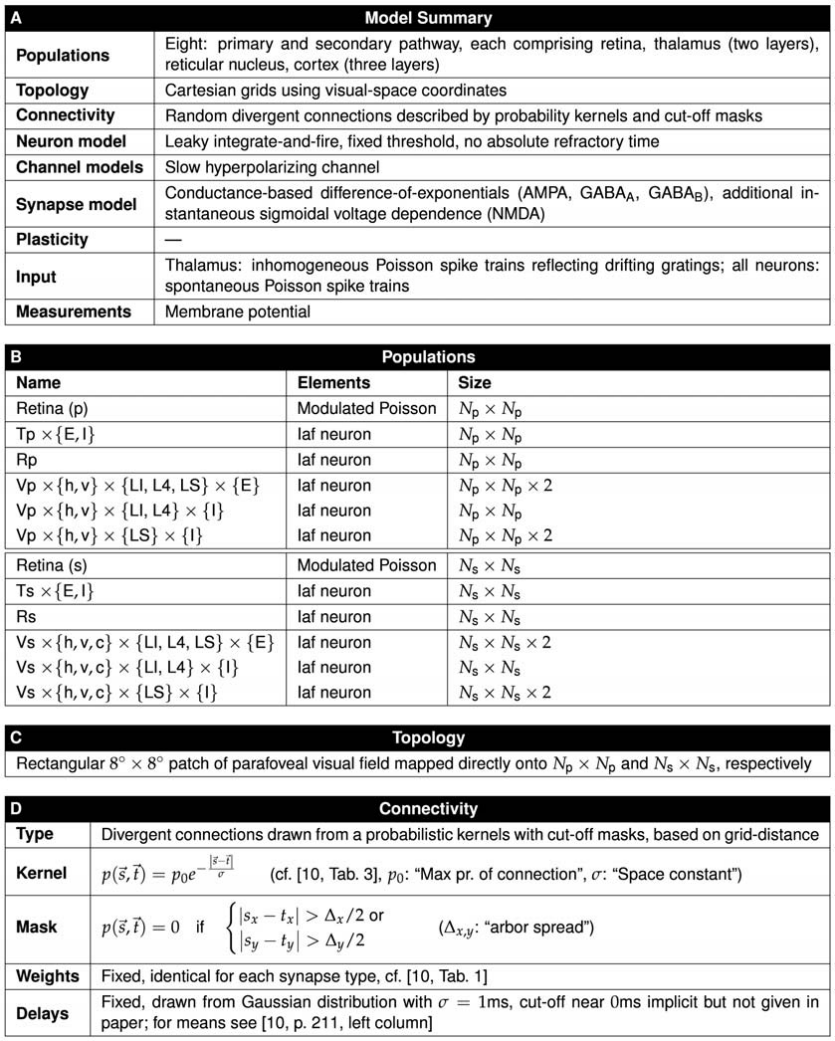
Tabular description of Lumer et al. model [10], part 1. The model is summarized in panel A and detailed in panels B–I. See Figure 8 for panels E–I.

We will discuss these elements in turn below, followed by more detailed discussions about how to render specific aspects of a network model. As an illustrative example, Figures 5 and 6 provide a concise description of the Brunel [3] model following the good practice format. A similar description of the Lumer et al. [10] model is given in Figures 7–9.

**Figure 8 pcbi-1000456-g008:**
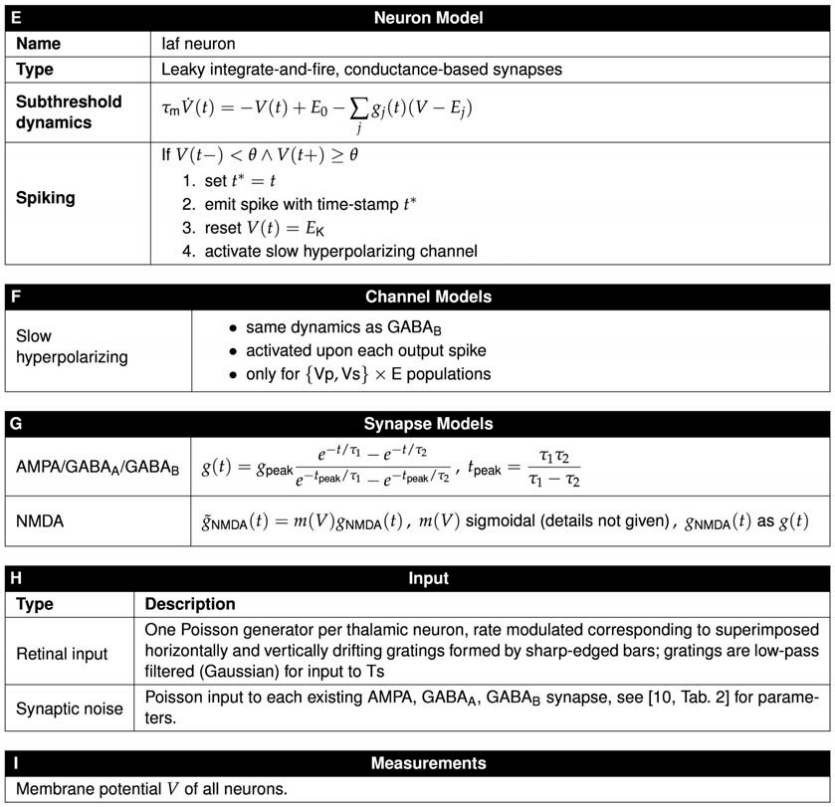
Tabular description Lumer et al. model [10], part 2. See Figure 7 for panels A–D.

**Figure 9:**
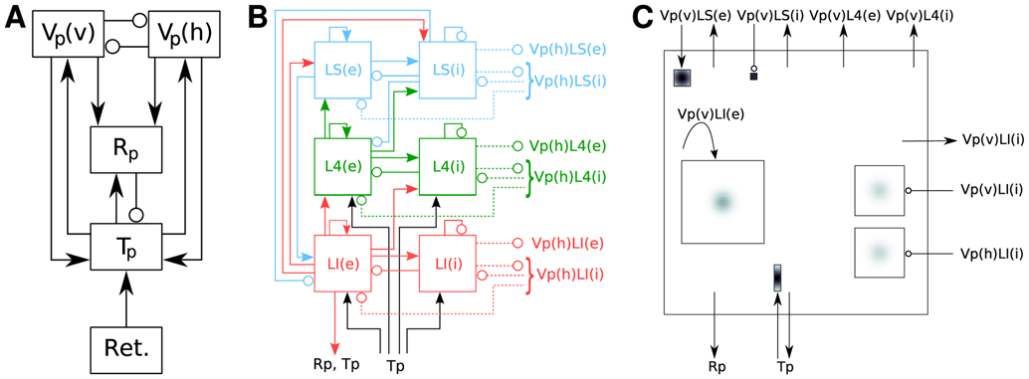
Hierarchy of diagrams of a complex network model (Lumer et al. [10]). (A) Overview diagram of connectivity between high-level populations. Excitatory connections are marked by arrows, inhibitory connections by circles. Excitatory and inhibitory populations have been lumped in Tp, while Vp(v) and Vp(h) are composed of three layers of excitatory and inhibitory populations, as detailed in B. (B) Detailed diagram of connectivity within cortical population Vp(v), which is tuned to vertically oriented stimuli. Vp(v) is composed of three cortical layers, each with an excitatory (left) and inhibitory (right) subpopulation. Filled arrows mark excitatory, open circles inhibitory connections. Connections to and from corresponding horizontally tuned cortical populations in Vp(h) are shown as dashed lines; black lines show input from the thalamus. Connections to and from higher cortical areas are not shown. (C) Detailed rendition of connection masks and kernels projecting onto one cortical subpopulation Vp(v)LI(e) from panel B, i.e., the excitatory subpopulation of the infragranular layer of Vp(v). Squares show projection masks, gray shade the probability of a connection (black: 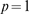). Connections are created by centering the mask about each location in the layer and drawing connections according to the probability distribution. Outgoing arrows indicate projections to other populations. Projection masks are scaled down in size to fit all projections into the layer, and grayscales have been adjusted for visibility. Connections are placed to correspond to the layout of panel B: Connections to and from thalamus are at the bottom, connections to and from Vp(v)LI(i) and Vp(h) to the right and connections to and from Vp(v)LS and Vp(v)L4 at the top.

We would like to stress that we present the good practice here to stimulate the debate on model descriptions within the computational neuroscience community. If it is adopted widely throughout the community, it will provide numerous advantages: authors will have guidelines that will allow them to check their descriptions for completeness and unambiguousness; referees will more easily be able to assess the correctness and quality of a model; and readers will find it easier to comprehend and re-implement a model, and to compare different models.

#### Guidelines for organization

Many journals require authors to organize their manuscript into the sections *Introduction*, *Results*, *Methods*, and *Discussion* and the question arises how modeling papers fit into this framework. We believe that this organization is also appropriate for modeling papers if the meaning of the individual section headings are carefully observed.

Generally, a publication on a computational modeling study should provide the following information:

1. *Hypothesis:* a concrete description of the question or problem that the model addresses;
2. *Model derivation:* a presentation of experimental data that support your hypothesis, your model, or both;
3. *Model description:* a description of your model, its inputs (stimuli), and its outputs (measured quantities) and all free parameters, according to the good practice proposed below;
4. *Implementation:* a concise description of the methods used to implement and simulate the model (e.g., details of spike threshold detection, assignment of spike times, time resolution, etc.), as well as a description of all third party tools used, such as simulation software or mathematical packages;
5. *Model analysis:* a description of all analytical and numerical experiments performed on the model, and the results obtained;
6. *Model justification:* a presentation of all empirical or theoretical results from the literature that support the results obtained from your model and that were not used to derive the model.

We suggest that authors organize their presentation according to these six points where possible. When publishing in a journal that requires a traditional organization of manuscripts into *Introduction*, *Results*, *Methods*, and *Discussion*, we recommend the following structure:

1. *Introduction*
  - *Hypothesis*
  - *Model derivation*
2. *Results*
  - *Model description*
  - *Model analysis*
3. *Methods*
  - *Implementation*
4. *Discussion*
  - *Model justification*

The paper should be written such that readers who are not interested in model derivation and implementation can skip these sections to proceed directly from the model description to the analysis.

Many journals impose strict limits on the length of a paper, making it impossible to provide a full model description along with an elaborate model analysis. In this case, authors should consider to split their manuscript in two (or more) separate manuscripts: One describing the model, and the other describing the model analysis. The model paper should include the full description of the model but with the model analysis section reduced to only that information which is needed to validate the model and its implementation. In the analysis paper, authors can cite the model paper and reduce the model description to a brief outline of the model, using the tables proposed below. This should offer sufficient room to include a full account of the model analysis.

Where a companion paper is infeasible, authors should provide a detailed model description as online supplementary materials, although we see two disadvantages in this case: (i) Supplementary material might not be peer-reviewed according to the same high standards as a separate model paper. (ii) Hiding the model in supplementary material deprives both author and model of the proper credit for the intellectual effort that went into the creation of the model.

Authors should be encouraged to make their model implementation available through community repositories under suitable licensing terms [17],[67], to promote re-use. We expect professionally managed repositories for neuronal network models to emerge that will give equal weight to human comprehensible and machine readable model descriptions, and curate them according to precisely defined quality standards; such efforts are underway in a number of communities [37], [50], [68]–[70]. Once such a repository is firmly established for computational neuroscience, papers might reference detailed model descriptions in a repository, instead of including a full description in the paper itself.

#### Checklists for model descriptions

Model descriptions should give the reader a good overview of the overall structure of a model. We suggest a description in prose accompanied by figures. The text should give an introduction to each composite part, i.e., stating the number of parts, their size, and what sub-parts they consist of. We recommend that authors concisely summarize the information for each part in standardized tables (see panel A in Figures 5, 7, and 8) and quote only the most necessary pieces in the text. We will discuss network diagrams in detail below.

Following the principle that models should be presented *top-down*, we suggest that authors adhere to the following order when describing the parts of their models:

1. Model composition
2. Coordinate systems and topology
3. Connectivity
4. Neurons, synapses, and channels
5. Model input, output, and free parameters
6. Model validation
7. Model implementation

Not all parts will apply to all models, but using such a checklist (i) ensures that all necessary information is included in the paper; (ii) allows referees to systematically check that all information is given; and (iii), facilitates the comparison with other models. We will address each of the items in the list below.

Past experience indicates that it is essential to review model descriptions after one has implemented a model [32]. We strongly suggest that authors carefully compare model description and implementation. This ensures that the description is complete and that any choices made during implementation are duly reflected. If possible (and feasible), one should ask a colleague to re-implement the model based on the description.

The **model composition** are the groups, or populations, of neurons in a network model. Populations are either unordered, such as Brunel [3] and Haeusler and Maass [5], or ordered, such as the remaining models in Table 1.

A good model description should list all populations of the model along with the used neuron model, their properties, their number, and how each population relates to the modeled system. Authors should name each population and use this name consistently throughout the manuscript. Some populations may be selections of neurons from other populations. In this case, authors should give explicit selection rules or equations.

Even for random selections, we recommend that authors explicitly define the actual range of indices used, to avoid formulations such as “we recorded from 50 randomly selected neurons”, when indeed a contiguous range of 50 neurons from an unordered population was chosen [3].

**Coordinate systems and topologies** describe how individual neurons in a population can be addressed, or selected, and, where applicable, the spatial relationships between neurons. Authors should specify all coordinate systems used, because they are central to defining the connectivity of the network.

The most basic is the *index coordinate system* which numbers each neuron in the population. Index coordinates are often one-dimensional, but if the populations are representing sheets or volumes of nervous tissue, index coordinates may become two-, three-, or even higher dimensional. Index coordinates are *unordered*, because they do not imply a neighborhood relation between any two neurons, nor do they define a distance function (e.g., Brunel [3]).

Many models have additional coordinate systems, e.g., *anatomical coordinates*, if the coordinates within a population refer to positions in the brain, as in Izhikevich and Edelman [8], or *logical coordinates*, if the coordinates within a population refer to some logical property, such as stimulus dimensions or response properties, e.g., orientation angle, as in Lumer et al. [10]. Anatomical or logical coordinates impose a *topology* on the unordered population, because they allow one to measure distances between neurons.

For each coordinate system used, authors should state exactly how the coordinates are mapped to the index coordinates of the population. A good model description should also give explicit expressions for all distance functions used.

The description of the **connectivity** can now build on the defined populations and coordinate systems. To describe the connections we suggest using prose, equations and figure(s). Authors should start with an overview of the connectivity at the level of populations, followed by all information needed to link connectivity at the level of populations to the connections between individual neurons. The following checklist may assist authors in this task:

1. Are all populations of pre- and post-synaptic neurons defined?
2. Are all coordinate systems defined which are needed to select pre- and post-synaptic neurons?
3. How are pre-synaptic neurons selected from a population?
4. How are post-synaptic neurons selected from a population?
5. How are boundary effects in topological connections handled?
6. If a pair of pre- and post-synaptic neurons can be chosen more than once, is this connection allowed?
7. If the same neuron can be selected as pre- and post-synaptic neuron, is this connection allowed?
8. How are the parameters (e.g., weight and delay) of a connection determined?
9. If random connections are used, provide the algorithm used to select the pre- and post-synaptic neurons and to determine whether a connection is made.
10. Are all parameters of the connectivity explained and are their numerical values given?

A figure of the connections in addition to the textual description is of great help to the reader. Suggestions for how to draw connection diagrams are given below.

To describe the dynamics of **neurons, synapses, and channels** we suggest a combination of prose and equations. The text should give the overview, the equations the detail, since they are more exact.

It is important to describe how the neuron behaves over time. For spiking models, the description should encompass how the neuron behaves before, during and after a spike is generated, e.g., state the spike threshold, set the refractory period and define if there is a potential reset. Since this part of a neuron model is often algorithmic, pseudo-code or flow-charts may be an effective means of description. There should also be a description of the synapse type and its behavior, and the algorithms for the plasticity should be given.

**Model input, output, and free parameters** are important aspects of a model. Models in computational neuroscience mostly attempt to describe systems rather than phenomena. This is shown by the fact that none of the models we investigated explicitly states its input and output variables.

By contrast, models in statistics are built around the concepts of independent variables (stimulus), dependent variables (response), and the free parameters of a system. A model is then a function that maps the independent variables onto the dependent variables, using the free parameters. We find this view helpful, because it makes the scope of a model explicit.

We suggest that authors explicitly list the independent and dependent variables of their model, along with all free parameters. A textual description of the stimuli accompanied by tabulated parameter values will suffice in most cases to recreate the stimuli. In addition, readers will benefit from a figure illustrating non-trivial stimuli, such as Fig. 1 in Hayot and Tranchina [6]. If the model uses complex stimuli, such as images or sound sequences, authors should make them available online, so that readers can re-implement the model. A good model description should also detail how responses are measured.

The following checklist may help authors to compile all information for the model description. Most of this information is best placed in the tables, suggested below.

1. *Model input*
  - Describe the stimulus ensemble;
  - Describe which parts of the model are stimulated;
  - Describe exactly how the stimulus is applied;
  - Describe any scaling or normalization of the stimulus.
2. *Model output*
  - Describe which quantities are measured;
  - Describe exactly from which parts of the model measurements are taken;
  - Describe exactly how measurements are taken (e.g., specify the sampling rate of the measurements);
  - Describe how output quantities are computed from the measurements (e.g., firing rates from spike-trains).
3. *Free parameters*
  - Describe all free parameters of the model;
  - List the chosen values for each parameter.

**Model validation** is crucial to the reliability of modeling studies. Authors should provide information that will allow others to systematically test re-implementations of neuronal network models. To this end, they should include, e.g., membrane-potential traces of model neurons in response to current injection and crafted spike trains.

These figures help readers who attempt to re-implement a network model to validate their implementation of the neuron models; Destexhe et al. [4] and Izhikevich and Edelman [8] are fine examples in this respect. Unless the model is new, such figures are best placed in the appendix. For models that are well known in the literature, these figures may be put in the supplementary material.

Testing that parts of a model behave as expected is an excellent way of reducing the chance of errors at a later stage, and is also known as *unit testing* [71]. If performed in stages, unit testing ensures that all components at a given level function properly, such that any difficulties at the next level of integration can be localized to that level. Systems biologists are ahead of neuroscientists in this respect, and have addressed this issue through the development of the SBML Semantic Validation Suite [35].

Authors should specify the **model implementation**, i.e., list details of the tools and methods that were used to obtain numerical results. The information should be sufficient to allow readers to re-implement the model and its analysis.

The following list may assist authors in compiling the required information:

1. Which software was used to analyze the model?
  - If third party software was used, list the name, version, and provider of the software.
  - If self-written software was used, provide sufficient information on the algorithms and numerical methods used, to allow re-implementation.
  - Consider making the simulation program/scripts available as supplementary material.
2. Which parameters, such as integration stepsize and accuracy goals, were used?
3. Which software was used to analyze and visualize the data obtained from the model?
  - If third party software was used, list the name, version, and provider of the software.
  - If self-written software was used, provide sufficient information on the algorithms and numerical methods used, to allow re-implementation.
  - Consider making the simulation program/scripts available as supplementary material.
4. Consider making your analysis scripts available as supplementary material.

#### Templates for tables

To provide a full description of the network model, we encourage authors to detail each model part. Figures 5, 7, and 8 illustrate how such detailed descriptions may be given in concise form. We invite readers to use these tables as templates for their own publications.

At present, it does not seem possible, or even desirable, to define precisely how these tables should be formed. Indeed, the reader will notice that we describe the connectivity in the Lumer et al. [10] model in a rather different way than in the Brunel [3] model. Lacking any widely adopted formalism for the description of connections, we could at present not see any other way of providing descriptions that were at the same time compact and informative. The connection set algebra recently proposed by Djurfeldt [72] may eventually evolve into a common formalism for connectivity.

For now, we have set up our tables pragmatically as follows:

1. The first table shall always present a concise *Model Summary* based on the *Checklist* proposed above; one may compare it to the “Nutrition facts” box on food packaging. Non-applicable entries in the table shall be kept in the table to make explicit that a model does not have, e.g., topology or synaptic plasticity.
2. For each non-empty entry in the *Model Summary*, a table presenting details shall follow.
3. These detailed tables shall in themselves be concise and be presented in the same order as the entries in the *Model Summary*.
4. The tables shall contain the names (or symbols) used for populations, connections or other model elements in the modeling paper.
5. When model components have been obtained from a model repository, or have a precise definition in a relevant online ontology, accession numbers or ontology reference shall be given.

The tables proposed here describe the *structure* of the model. In addition, we propose that all *parameters* of a model should be given in tables to make them easily accessible; some authors do this already.

#### Guidelines for diagrams

Diagrams are a powerful way of expressing relations between parts of a model. Authors should use diagrams to illustrate their model structure and to specify relations between the different model parts. A good model description should use at least one diagram, showing the overall structure of the model. Further diagrams can then be given to elaborate on details and different aspects of the model.

Diagrams should be precise representations of a model and its parts. To this end, we must use the graphical vocabulary of shapes, lines and graphical styles to convey as much detail as possible without sacrificing clarity.

To achieve their full potential, diagrams need to follow a common standard, so that readers can perceive and compare diagrams from different publications. We have seen earlier that there are currently no established rules for drawing diagrams of neural network models. At this point, we give some tentative suggestions only, as sketched in Fig. 6 (Brunel [3] model) and Fig. 9 (Lumer et al. [10] model). These figures are based on the following principles:

1. Unordered populations are shown as circles;
2. Populations with spatial structure are shown as rectangles;
3. Pointed arrowheads represent excitatory, round ones inhibitory connections;
4. Arrows beginning/ending outside a population indicate that the arrows represent a set of connections with source/target neurons selected from the population;
5. Probabilistic connection patterns are shown as cut-off masks filled with connection probability as grayscale gradient; the pertaining arrows end on the outside of the mask.

We will return to the design of network diagrams in the discussion.

## Discussion

Communicating neuronal network models in scientific publications is a challenging task. We have demonstrated above that current publication practices are far from ideal. This has two unfortunate consequences: First of all, it hampers the critical, mutual assessment of published models. As a result, there is no tradition in the computational neuroscience community for scientists to cross-examine each others models thoroughly. The validation of models thus typically remains at the level of individual studies and publications, i.e., not as reliable as is desirable. Other fields, in contrast, have established the validity of their central models beyond any reasonable doubt—and with a clear understanding of their limits of viability—such as the central laws of classical and quantum mechanics, electrodynamics and statistical physics. A second unfortunate consequence of present publication practices is that neuronal network models are rarely re-used by others, thus reducing the overall productivity of the computational neuroscience community. This second consequence follows to a large degree from the first, as few scientists would like to re-use models unless their validity was properly established; in addition, the lack of precision in today's model descriptions often makes re-use difficult.

### Network diagrams

The model survey presented here revealed a wide variety of approaches to describing the composition and connectivity of neuronal networks. We believe that this is, at least in part, due to a lack of common high-level concepts for composition and connectivity from a modeling perspective. Developing such high-level concepts describing, e.g., certain types of randomized connectivity patterns, is thus an important task for the computational neuroscience community. The challenge at hand is perhaps best clarified when trying to draw diagrams representing neuronal network models. Such diagrams have two aims: To give the reader an intuitive understanding of model properties central to the dynamics of the model, and to unambiguously provide the necessary detail to allow a reconstruction of a model. In the absence of a mathematical formalism for model specification, diagrams often seem better suited than prose to present unambiguous detail. Simple models, such as that by Brunel [3], can be depicted in a single diagram, as illustrated in Fig. 6. The four panels in that figure, though, show that one may choose from a wide variety of styles for such diagrams, and it is not *a priori* clear which style is best. In panels A–C in the figure we propose three ways to differentiate between excitatory and inhibitory connections (line styles and endings) as well as to mark connectivity patterns (line endings, styles, annotations). Panel D differs from the other three in the way the external input is represented. Brunel [3] states that “[each neuron] receives 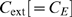 connections from excitatory neurons outside the network. External synapses are activated by independent Poisson processes with rate 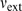.” This is rendered in detail in panel D, which shows 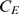 Poisson generators per modeled neuron. In all other panels, these generators have been collapsed into an external excitatory population 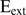 with the implicit assumption that this population contains the correct number of Poisson generators required by the model.

Presenting complex models is even more challenging. In Fig. 9, we present a set of three figures describing the model by Lumer et al. [10] at three levels of hierarchy: an overall view in panel A, details of the connectivity within the cortical populations tuned to vertical stimuli in panel B, and finally details of projection patterns into a single cortical population in panel C. All figures are simplifications of the full model, since we have left out the secondary thalamic and cortical areas. We are currently pursuing research to identify drawing styles and a hierarchy of diagrams that will be intuitive to a majority of computational neuroscientists and provide the necessary detail. Results will be presented elsewhere.

### Why are standards lacking?

Given the importance of comprehensible and precise model descriptions, it may seem surprising that no standards or good practices have emerged in computational neuroscience to date. Early proposals, such as the Neural Simulation Language [73] (see also Eliasmith and Anderson [49], Ch. 1.5 and Kumar [74]), have not been accepted widely in the community.

At present, two developments appear promising. NetworkML, which is part of the NeuroML project [28],[51], provides a simulator-agnostic XML-based declarative standard for neuron network model descriptions. Simulation code for tools such as Neuron and Genesis can be generated from models defined in NeuroML. PyNN [29], in contrast, is an imperative scripting language that can control a number of common neuronal network simulators, such as NEST, Neuron, and Brian. One reason why neither NetworkML nor PyNN has yet caught on as a means of widespread model exchange may be that neither of the two languages seems to aim at providing human-comprehensible model descriptions that might be included in publications.

Another reason for the lack of model description standards may be that computational neuroscience has to a large degree been an ancillary science, an appendix of electrophysiology: The vast majority of publications in computational neuroscience compares its modeling results directly to specific sets of experimental data. And even though models have driven the development in some fields of neuroscience [75], very few authors have compared the properties of different models with each other; Erwin et al. [76] is a notable exception. De Schutter [77] even argues that there currently is a trend away from the investigation of models as such, and back to a one-to-one matching of models to experiments. As long as computational neuroscientists focus on matching their models to specific experiments, rather than either to spar their models against each other, or build their models upon each other, the motivation to use a standard notation is obviously limited.

### Perspectives

We have no doubt that model sharing will increase in computational neuroscience in years to come. This raises the question of what model sharing precisely entails. At the simplest level, models may be shared as simulator code. While this seems convenient at first, it carries significant risk, as any code is likely to contain errors, in particular errors that may surface only once an existing model is used in a different context than the one in which it was originally developed. Indeed, in at least one case, high-profile publications (outside neuroscience), had to be retracted after a subtle programming error was discovered in a widely shared scientific software [78]. Some scientists argue that everyone in a field should use the same, carefully maintained simulation software to avoid such problems, and to make computational science reliable [79]. We beg to differ: monoculture tends to create more problems than it solves.

Establishing a new publication culture in computational neuroscience will require considerable effort within the community. We hope that the *good model description practice* that we have outlined in the previous section may be a good starting point. We believe in particular that a clear segregation of model derivation, model description, implementation, and model analysis, as proposed above, will make it easier for readers to discern the model as such, compare it to other models, and evaluate its relevance to their own research. The proposed *Checklists for model descriptions* will help to ensure that model descriptions themselves are reasonably complete and follow a common pattern, further improving the communication of models, while the *Templates for tables* invite a standardized presentation of details on various aspects of models; similarly, the *Guidelines for diagrams* should aid authors in illustrating their network models. Since all our proposals are informal, we hope that authors will find it straightforward to apply them when describing their network models, thus establishing a *de facto* standard for model descriptions.

We are optimistic that we are beginning to see changes towards more cooperation within computational neuroscience, as witnessed by several collaborative reports on neuronal network simulations in the last two years [25]–[27] and the development of tools for the integration of various simulation software [29],[30], much helped by the establishment of the International Neuroinformatics Coordinating Facility (INCF) in 2005. The Connection Set Algebra proposed by Djurfeldt [72] is an encouraging step towards establishing high-level concepts for neuronal network descriptions, i.e., giving us a concise language to talk about our models. There is also much to be learned from model sharing and curation efforts in other communities, such as the IUPS *Physiome* and the European *Virtual physiological human* projects [37],[80].

In closing, let us return to the power of notation, as exemplified by the matrix notation in the introduction. In July 1924, Werner Heisenberg gave a manuscript full of complicated mathematics to his mentor Max Born, unsure whether it was worth publishing. Born worked through Heisenberg's ideas and realized that what Heisenberg had written down, actually amounted to the matrix mechanics of quantum theory. This insight of Born's unleashed the full power of Heisenberg's ideas and let Born discover the non-commutativity of quantum mechanics [81], p. 125f. We are looking forward to the day when a good formalism will give us deeper insights into the secrets of signal processing in the brain.

## Supporting Information

Table S1 Network architecture description: placement and means. Each table entry gives the number of papers from Table 1 in main paper using a given means (columns) and location (rows) to describe the network architecture of the model used, with row- and column-wise totals to the right and at the bottom. Most papers combine several modes of description; the “References”-column contains papers that do not give explicit descriptions, but point to published models. The network architecture description is an overview only, and details are left out. That is the reason for why columns “Eqns” and “Tables” are empty here. See Table 1 in main paper for paper abbreviations.
(0.27 MB PDF)

Table S2 Network connectivity description: placement and means. The presentation is the same as in Table S1.
(0.10 MB PDF)

Table S3 Neuron and synapse model description: placement and means. The presentation is the same as in Table S1.
(0.12 MB PDF)
