# Supplementary material for: Towards Reproducible Descriptions of Neuronal Network Models
Source: PLoS Comput Biol. 2009 Aug 7;5(8):e1000456. doi: 10.1371/journal.pcbi.1000456 (PMC2713426; doi:10.1371/journal.pcbi.1000456)
Supplement: Table S1 — Network architecture description: placement and means. Each table entry gives the number of papers from Table 1 in main paper using a given means (columns) and location (rows) to describe the network architecture of the model used, with row- and column-wise totals to the right and at the bottom. Most papers combine several modes of description; the “References”-column contains papers that do not give explicit descriptions, but point to published models. The network architecture description is an overview only, and details are left out. That is the reason for why columns “Eqns” and “Tables” are empty here. See Table 1 in main paper for paper abbreviations. (0.27 MB PDF) [file pcbi.1000456.s001.pdf]

	Prose	Eqns.	Figures	Tables	Refs.	Total
**Paper**	B, D, HM, HT, HvH, IE, KG, L, M, SE, TA, TR, VA, WS		HM, HT, HvH, IE, KG, L, VA		WS	22
**Appendix**	HT					1
**Supplement**	IE, M, TA		IE			4
**Total**	18	0	8	0	1	27

Table S1: Network architecture description: placement and means. Each table entry gives the number of papers from Table 1 in main paper using a given means (columns) and location (rows) to describe the network architecture of the model used, with row- and column-wise totals to the right and at the bottom. Most papers combine several modes of description; the "References"-column contains papers that do not give explicit descriptions, but point to published models. The network architecture description is an overview only, and details are left out. That is the reason for why columns "Eqns" and "Tables" are empty here. See Table 1 in main paper for paper abbreviations.
