# Supplementary material for: Towards Reproducible Descriptions of Neuronal Network Models
Source: PLoS Comput Biol. 2009 Aug 7;5(8):e1000456. doi: 10.1371/journal.pcbi.1000456 (PMC2713426; doi:10.1371/journal.pcbi.1000456)
Supplement: Table S2 — Network connectivity description: placement and means. The presentation is the same as in Table S1. (0.10 MB PDF) [file pcbi.1000456.s002.pdf]

	Prose	Eqns.	Figures	Tables	Refs.	Total
**Paper**	B, D, HM, HT, HvH, IE, KG, L, M, SE, TA, TR, VA	B, HvH, TR, WS	D, HM, HvH, IE, KG, SE, TA, TR	SE	WS	27
**Appendix**	HT, L	HT		L		4
**Supplement**	IE, M, TA	IE, M	IE	IE		7
**Total**	18	7	9	3	1	38

Table S2: Network connectivity description: placement and means. The presentation is the same as in Table S1.
