# Supplementary material for: Towards Reproducible Descriptions of Neuronal Network Models
Source: PLoS Comput Biol. 2009 Aug 7;5(8):e1000456. doi: 10.1371/journal.pcbi.1000456 (PMC2713426; doi:10.1371/journal.pcbi.1000456)
Supplement: Table S3 — Neuron and synapse model description: placement and means. The presentation is the same as in Table S1. (0.12 MB PDF) [file pcbi.1000456.s003.pdf]

	Prose	Eqns.	Figures	Tables	Refs.	Total
**Paper**	B, D, HM, HT, HvH, IE, KG, L, M, SE, TA, TR, VA, WS	B, D, HM, HvH, IE, L, M, SE, TA, TR, VA, WS	D, HM, IE, TA	HM, L, SE	D, HM, HvH, TR, WS	35
**Appendix**	B, HT, KG	B, HT, KG				6
**Supplement**	IE, M, TA	IE, M, TA		IE		7
**Total**	20	18	4	4	2	48

Table S3: Neuron and synapse model description: placement and means. The presentation is the same as in Table S1.
